# Cuffless Blood Pressure Estimation Based on Data-Oriented Continuous Health Monitoring System

**DOI:** 10.1155/2017/1803485

**Published:** 2017-04-24

**Authors:** Kengo Atomi, Haruki Kawanaka, Md. Shoaib Bhuiyan, Koji Oguri

**Affiliations:** ^1^Graduate School of Information Science and Technology, Aichi Prefectural University, Aichi, Japan; ^2^Faculty of Medical Engineering, Suzuka University of Medical Science, Mie, Japan

## Abstract

Measuring blood pressure continuously helps monitor health and also prevent lifestyle related diseases to extend the expectancy of healthy life. Blood pressure, which is nowadays used for monitoring patient, is one of the most useful indexes for prevention of lifestyle related diseases such as hypertension. However, continuously monitoring the blood pressure is unrealistic because of discomfort caused by the tightening of a cuff belt. We have earlier researched the data-oriented blood pressure estimation without using a cuff. Remarkably, our blood pressure estimation method only uses a photoplethysmograph sensor. Therefore, the application is flexible for sensor locations and measuring situations. In this paper, we describe the implementation of our estimation method, the launch of a cloud system which can collect and manage blood pressure data measured by a wristwatch-type photoplethysmograph sensor, and the construction of our applications to visualize life-log data including the time-series data of blood pressure.

## 1. Introduction

Holter electrocardiogram and SpO_2_ measurement are available as a monitoring method in daily life. Holter electrocardiogram can be used for diagnosis about the pacemaker adaptation and for detection of transient arrhythmia [1, 2]. The SpO_2_ measurement monitors the oxygen saturation in the blood percutaneously by a pulse oximeter equipped to a fingertip. The SpO_2_ measurement can be used for prognostic prediction of respiratory disease and for severity diagnosis of idiopathic pulmonary fibrosis [3].

In the same manner, the blood pressure (BP) monitoring has been receiving attention from the viewpoint of lifestyle disease prevention. By measuring the BP while undergoing normal daily life, the realization of diagnosis of nocturnal hypertension and morning hypertension, which are difficult to detect at the hospital setting, is expected. In a previous study, Noda et al. classified the nighttime blood pressure variation into 4 patterns using average BPM. They showed the importance of nighttime blood pressure variation [4]. Kario et al. had pointed out the necessity of full-time BP monitoring [5] since the risk of organ derangement is high in case that the BP rises or does not fall while sleeping as a circadian variation. However, continuous measurement using a cuff is difficult. Murohara et al. pointed out that using a cuff caused discomfort by tightening of a cuff belt. And then they have also expressed a necessity of BP measuring method without a cuff. Furthermore, like BP, it pointed out that accumulating lifestyle habit data such as exercise and meal could be useful for treatment and prevention of high blood pressure.

For continuous monitoring, method of blood pressure estimation from biomedical signals without cuff is being studied. Chen et al. measured BP and pulse wave by noninvasive method [6]. Then, they estimated BP based on PTT obtained from photoplethysmograph (PPG) and ECG. However, it is necessary to correct the estimated value every 5 minutes using the measured BP. Zhang et al. estimated BP using wrist-watch type electrocardiogram sensor and finger PPG sensor [7, 8]. Espina et al. put the electrocardiogram sensor on the waist and the PPG sensor on the ear and calculated the PTT [9]. In addition, they aimed to monitor blood pressure for a long time by acquiring and analyzing data using wireless communication. Zakrzewski et al. used ultrasound for noninvasive and nonocclusive BP estimation [10]. BP estimation method using PTT needs multiple sensors. It is considered desirable to use a single sensor in order to perform simpler measurements. Suzuki and Oguri used only a PPG signal to estimate BP by linear multiple regression analysis (PPG method) [11]. By applying the estimation method, Kawanaka et al. obtained a pulse wave (PW) from a fingertip using a PW sensor mounted on smart phones and estimated BP on a client server system [12]. It has shown that monitoring of BP without using a cuff is possible at any time or place if we can access an internet server with a smartphone device. The system, however, is considered to be unsuitable for continuous monitoring because a user must place a finger on the sensor of PW with the intention to measure every time. Based on the above, we thought that we need to develop a system that can continuously estimate blood pressure at measurement positions that do not interfere with work. And, toward realization of BP monitoring system, we need to implement system to collect and manage life-log data.

In this paper, we propose a cuffless BP estimation method using a wristwatch-type PPG sensor to realize the continuous BP monitoring. And we launched a cloud-based system in order to collect and manage life-log to a database on the Internet. In addition, we constructed applications that can visualize life-log data toward user clients and to the medical staff.

## 2. Blood Pressure Estimation from PPG

### 2.1. Photoplethysmograph and Acceleration Plethysmogram

The cuffless blood pressure (BP) estimation method generally uses pulse transit time (PTT) and pulse wave (PW) features extracted from the PPG. The method using PTT requires multiple sensors such as the ECG and the PPG to calculate PTT. Also, BP calibration that decides the initial value for BP variability is done beforehand by measuring with a cuff because it cannot estimate the absolute value of the blood pressure. Use of such multiple sensors and calibrating manipulation can make a user conscious of BP measurement, s/he may feel nervous, and this may lead to unnatural measurements. Ultimately, it is difficult to measure ordinary BPs in the same manner as of the white coat hypertension. Besides, as consciousness of measurement disrupts user's sleep, it is also difficult to measure correct BP data while sleeping.

We have earlier researched the measurement of BP without a cuff. In particular, an application as our research product is flexible for sensor locations and can make it calibration-free because our method of the BP estimation only uses a PPG sensor. Therefore, in this paper, we estimate BP using PPG sensor only [11].

**Figure 1:**
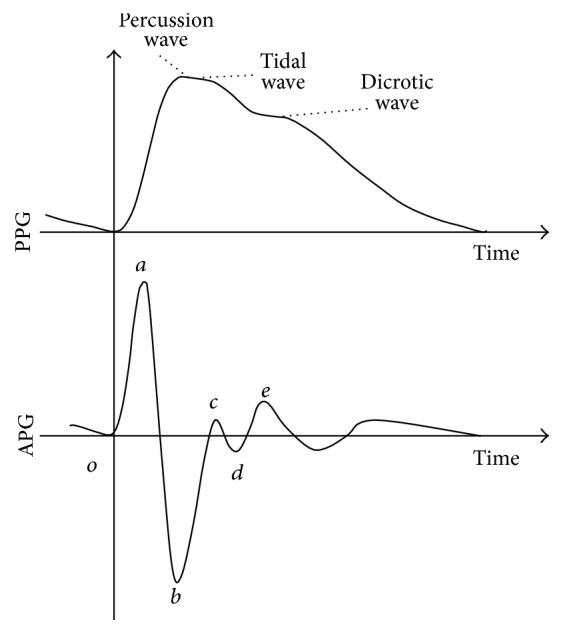
PPG, APG, and the systolic anterior components.

The second-order differential of PPG signal is defined as the acceleration plethysmogram (APG). An example of the waveforms of PPG and APG is shown in Figure 1. The horizontal axis is time, and the vertical axis is the wave height (signal intensity) of PPG and APG. The* a*-wave,* b*-wave,* c*-wave,* d*-wave, and* e*-wave are extracted as APG's peaks in chronological order. The* a*-wave and the* b*-wave are systolic anterior components which mean the driving pressure wave caused by blood ejection. The* c*-wave and the* d*-wave are systolic posterior components which mean the reflection pressure wave where the driving pressure wave was propagated in the periphery and then came back by reflecting. The* e*-wave is a diastolic component which means the peripheral blood flow after aortic valve closure. The waveform of PPG varies with blood vessel change. For example, elderly people tend to have stronger tidal waves than percussion waves in PPG [13]. Moreover, elderly people tend to have longer time from the start to the peak of PPG than young healthy people [14].

### 2.2. Features of Blood Pressure Estimation

As shown in Table 1, features are extracted from PPG, APG, and a preliminary questionnaire. In APG wave, features* a*,* b*,* c*,* d*, and* e* represent* a*-wave height,* b*-wave height,* c*-wave height,* d*-wave height, and* e*-wave height, respectively. And features* T*_*a*_,* T*_*b*_,* T*_*c*_,* T*_*d*_, and* T*_*e*_ represent the times elapsing from the rise of APG to* a*-wave,* b*-wave,* c*-wave,* d*-wave, and* e*-wave, respectively.

When a systolic blood pressure increases, the* a*-wave of APG tends to rise and the* b*-wave and* e*-wave decrease. Inversely, when a diastolic blood pressure increases, the* a*-wave tends to decrease and the* b*-wave and* e*-wave rise. And, APG is influenced by the elastic modulus of arteriolar. Therefore,* a*-waves tend to increase with decreasing of the elastic modulus; inversely,* b*-wave and* e*-wave tend to decrease [15].

The ratio of each component wave height to a-wave height represents a state of the blood vessel. The* b*/*a* represents the extensibility of blood vessels; it increases with increasing age. The* c*/*a* decreases with increase in age. The* d*/*a* becomes small when reflected wave of the vessel is large. The* e*/*a* shows softness of blood vessels; it becomes large for young people where diastolic wave is clear [16, 17].

The APG index which is a comprehensive index of APG shows closer relationship with blood vessel age. These PPG features are varied depending on softness of blood vessels. And BP is related to softness of blood vessels which are adjusted by automatic nerve. That is, a variation of PPG features means a variation of BP [18, 19]. In addition, pulse rate is counted from the PW for the past one minute. Individual information, height, weight, age, and sex are also used for BP estimation. Height, weight, age, and sex (male: 0 or female: 1) were acquired as absolute value from the preliminary questionnaire. BP largely depends on softness of blood vessels, and the softness of blood vessels is related to age. For example, blood vessels become sclerotic with advancing age. And the characteristic of sclerosis differs according to sex. Increasing and decreasing of peripheral vascular resistance by the autonomic nervous system appear to PPG features. Therefore, our BP estimation is realized with static parameters such as age, sex, and weight and dynamic variable parameters such as pulse wave features.

### 2.3. Blood Pressure Estimation Method

**Figure 2:**
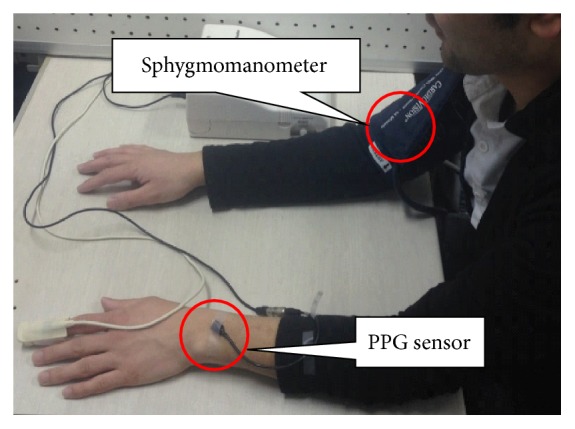
Blood pressure measuring environment using a PPG sensor and a sphygmomanometer.

In previous method, we researched a cuffless BP estimation method, which was a data-oriented approach, using huge amount of gathered data and machine learning algorithms [11, 12, 20, 21]. The measurement environment is shown in Figure 2. True BP, our target of estimation in this paper, was measured by a sphygmomanometer by fastening a cuff belt at the right upper arm. The subject as shown in Figure 2 was resting in a seating position while being measured. We extracted features from each PPG and APG which was measured by a PPG sensor placed at the left wrist. This dataset, which consists of BP value and several features, was used for the training of machine learning algorithm.

To derive an equation for BP estimation, we selected the multiple regression analysis as machine learning algorithm. We derived a regression equation based on the relationship between features and BP. The regression equation is formed as follows:

(1) $$\begin{array}{c} y=u_{0}+u_{1}x_{1}+u_{2}x_{2}+\cdots +u_{p}x_{p}, \end{array}$$

where *y* is the objective variable which corresponds to BP, *x*_*i*_  (*i* = 1, 2,…, *p*) is the explanatory variables, *p* is the number of features, *u*_*i*_  (*i* = 1, 2, …, *p*) is the coefficient for *x*_*i*_, and *u*_0_ is the intercept. Every *u*_*i*_ is optimized by the stepwise selection using the collected data. Finally, BP can be estimated by assigning the features obtained from subjects to the constructed regression equation.

## 3. Construction of Cloud System for Monitoring

### 3.1. Cloud System for Monitoring

We propose to comprehensively deal with BP and life-log data using a cloud system. Microsoft Azure is used in the virtual server. A computer for management is connected to the virtual private network (VPN), and any user's terminals (e.g., smartphone) are allowed to access the cloud server by using HTTP. The way to access the server is limited to HTTP and VPN connection.

By using this cloud system, all the users can collect and manage their own life-log data and are not limited to any place and time. In addition, by using virtual server, user and administrator do not have to be limited by the data capacity of their terminals. By building a server on the cloud, the administrator of this cloud system can flexibly change some resource to fit the treated data size. Furthermore, by analyzing the huge dataset of the various users, medical staff can provide appropriate information of intervention based on the analysis of the result to the user as secondary information toward improvement of BP.

### 3.2. BP Estimation System by Wristwatch-Type PPG Sensor

If photoplethysmograph (PPG) is acquired from a sensor that is placed on the fingertip, it is difficult to do some work with a finger.

**Figure 3:**
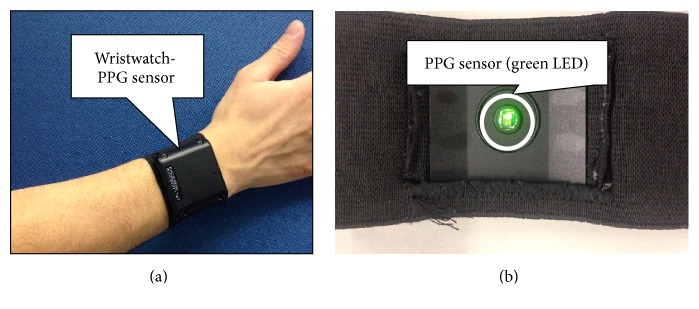
Wristwatch-type PPG sensor.

So, we focused on obtaining PPG from the wrist. To obtain PPG from the wrist, we used a wristwatch-type PPG sensor made by Denso Corporation.  Figure 3(a) shows a sensor mounted on the wrist.  Figure 3(b) shows the backside of the sensor. There is Green LED and photo detectors at the center of the sensor, and then it is possible to obtain PPG of the wrist. The PPG sensor's sampling rate was set at 100 Hz.

**Figure 4:**
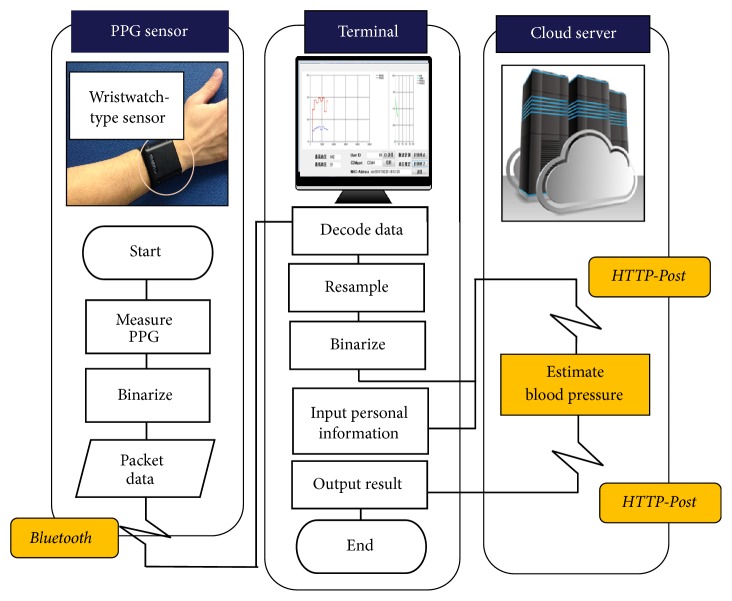
Flow chart of blood pressure monitoring system.

A flow chart of our BP monitoring system is shown in Figure 4. In this study, BP estimation system is implemented on the cloud server. By implementing on the cloud, we considered that a huge amount of PPG data will be accumulated. The stored PPG data can be utilized to estimate the health state in addition to BP. And we can utilize it to improve accuracy of BP estimation.

At first, a waveform signal obtained from a wristwatch-type PPG sensor is transmitted to a recording terminal (e.g., personal computer) via Bluetooth low energy. After receiving the waveform signal, the terminal sends a pulse wave (PW) data to the cloud server by using HTTP-Post. Then, the cloud server extracts features from the PPG and APG data and estimates BP. After that, the cloud server sends back the estimated BP to the terminal by using HTTP-Post, and it stores PPG data and the estimated BP data into database which are used to be viewed online by users and the medical staff.

### 3.3. Life-Log Recording Application

We have created a smartphone application to manage daily data (sleep, physical activity, and diet). The smartphone application performs information communication with the cloud server. The recording information on the cloud server is shown in Table 2. Measurement items are related to blood pressure.

For example, weight has positive relationship with BP [22]. By recording a meal, it is possible to relate the user's blood pressure and eating habits. Since it is difficult to record all the ingredients contained in each meal, meal contents are recorded by a meal image in this study. By collecting meal images, we can know the user's approximate salt intake. Salt intake correlates positively with blood pressure [23–25], and knowing salt intake is considered to be effective for hypertension treatment [26]. Also, alcohol intake is related to blood pressure. The higher the alcohol intake, the higher the possibility of high blood pressure [27–29].

It is considered that the user's physical activity level can be known from the acceleration. In particular, the difference in physical activity between sleeping and awake time is said to determine the degree of dipping [30]. Bedtime hour and awaking time hour can know the user's life rhythm. Midnight workers are said to have a long time of high blood pressure, and it is said that the frequency of high blood pressure increases in 24 hours [31–33].

From recording date of events, we can know the specific season when the data was. It is shown that blood pressure will rise in the cold season [34–37]. It is also pointed out that cardiovascular diseases are associated with seasonal variation [38].

Each user registers and then inputs a system ID in the application. The recording life-log and BP data is associated with ID assigned to each user. All data is sent as an XML format file from smartphone to access the cloud server by using HTTP-Post. Before sending data to the cloud server, the smartphone application inserts it into SQLite database that is constructed inside the application on the supposition of communication failure. So, the data is temporarily held even if it cannot be sent. After the communication recovery, data is sent again to the cloud server and stored in the database of the cloud server.

### 3.4. Visualization for Application Users

**Figure 5:**
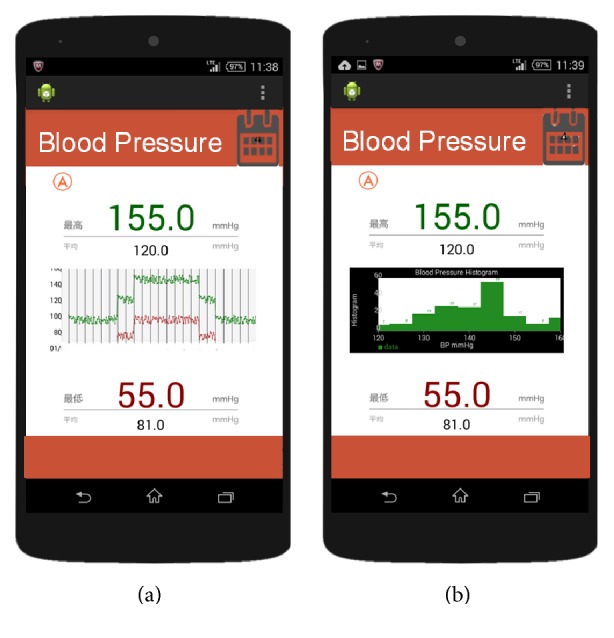
Visualization of blood pressure variation and histogram.

We developed a visualization application to monitor BP continuously. On the basis that the application will be used by smartphones, we developed the interface using Android in the same way as life-log recording. In Table 3, functions of the application are summarized. As shown in Figure 5(a), this application can display BPV (line graph), average of SBP, average of DBP, maximum of SBP, and minimum of DBP for the day.

It can plot a blood pressure variation (BPV) for one day using a line graph and display a pattern classified from the 24 hours continuous BP according to BPV pattern classification [39] proposed by Bando et al. They have defined the BPV patterns as follows. “Nondipper” means a pattern with nocturnal decrease of 10% or less than daytime averages of systolic blood pressure (SBP) and diastolic blood pressure (DBP). Here, daytime means from 6 am to 10 pm and nighttime means from 10 pm to 6 am. “Dipper” means a nocturnal decrease of 10% or more compared to daytime averages. “Extreme-dipper” means a nocturnal decrease of 20% more compared to daytime averages. “Riser” means a nocturnal increase compared to daytime averages.

In Figure 5(b), a picture of the screen of statistical information of the obtained data is shown, visualization of BP Histogram. By plotting a histogram of BP measured for a certain period, application users can acquire information of BP distribution. Furthermore, by displaying the maximum BP and minimum BP for each day, we come to know the day in which BP is higher or lower. It is effective also in observing the periodicity of one-week BPV.

The onset of cardiovascular disease differs by the day of the week. In particular, onset frequency is high on Monday [40, 41]. In-week variation is confirmed early in the morning and in the daytime [42]. It is thought that BP statistics can easily obtain data on in-week variation.

### 3.5. Visualization for Medical Staff

**Figure 6:**
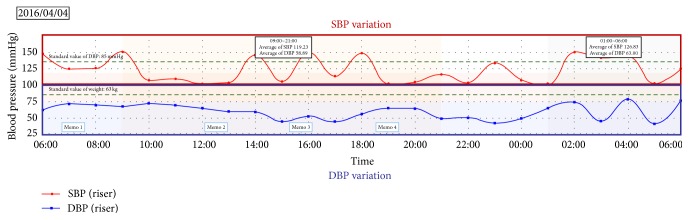
Visualization of blood pressure variation.

We developed a web application to provide information obtained from life-log data to medical staff. As shown in Figure 6, this application can display SBP variation and DBP variation. This application can display BPV for one day, one week, or one month.

**Figure 7:**
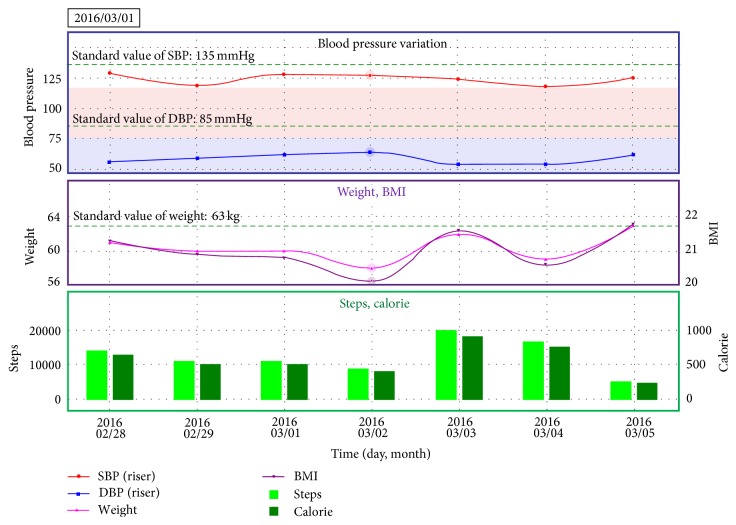
Visualization of BP, weight, and pedometer variation for one week.

However, it is difficult to provide appropriate guidance to patients by BPV only. Therefore, we thought that displaying simultaneously not only blood pressure but also life-log data will lead to appropriate guidance. By displaying the life-log data and the BPV at the same time, it would be possible to know the cause of the change in the blood pressure. As shown in Figure 7, this application can display BP, weight, and pedometer at the same time. In addition, BMI is calculated from height and weight, and calorie consumption is calculated from the pedometer. Average value per 24 hours for one week or average value per 30 days for one month can be calculated and displayed. Because the application is able to display a pattern of daily BPV trend [11] in the same way of visualization application for users, medical staff can know BPV pattern of patients.

## 4. Accuracy Verification of BP Estimation

**Figure 8:**
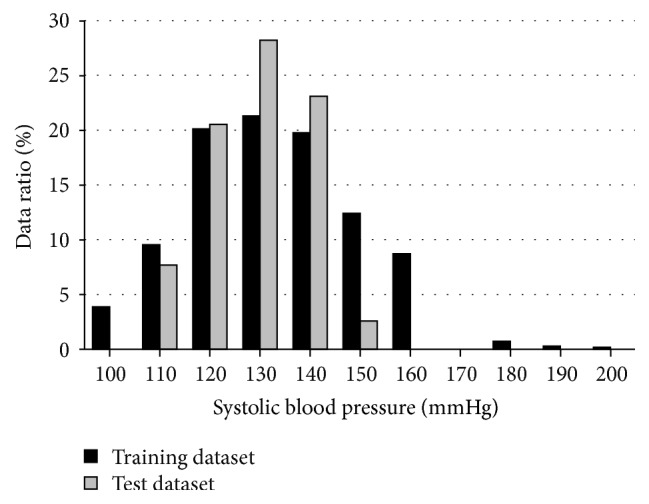
SBP distribution of training and test dataset.

We verified the accuracy of the blood pressure estimation equation introduced to the cloud system by experiment. For the training dataset used for constructing the regression equation, we collected 632 datasets of SBP and PPG features. SBP was measured by a sphygmomanometer using a cuff at the right upper arm, and the PPG was measured by a photoplethysmograph (PPG) sensor placed at the left wrist. The average age of subjects in the training dataset was 67.0 ± 8.8, and SBP was 127.6 ± 18 mmHg of which the SBP distribution is shown in Figure 8. Features selected by the stepwise multiple regression analysis were PR,* b*,* T*_*c*_,* c*,* d*, height, weight, and age from features listed in Table 1.

**Figure 9:**
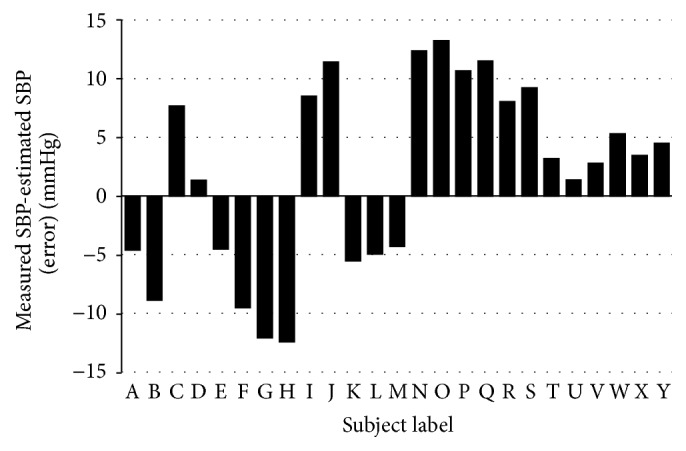
Mean error for each subject.

Next, in order to evaluate estimation accuracy, we conducted measurements for test dataset. 25 subjects had their PW measured using the wristwatch-type PPG sensor every minute for a total of 5 minutes while in seating position. Thereby, 122 sets of data were obtained after eliminating troubles in measuring. The average age of the subjects in the test dataset is 22.7 ± 1.2, and their average SBP is 116.0 ± 13.4 mmHg as shown in Figure 8. Regarding an estimation of the accuracy of our method, the correlation coefficient between the measured SBP and the estimated SBP was 0.80. The mean error was 1.58 mmHg, and the standard deviation of errors was 8.54 mmHg. Mean error and the standard deviation of errors for each subject are shown in Figure 9. The horizontal axis is the ID of the test subjects, the vertical axis is the mean error, and a black line on the bar graph is the standard deviation of errors.

## 5. Accuracy Verification of BPV Estimation

Our method does not estimate the BP fluctuation amount but can directly estimate the absolute value of BP at rest as with a general sphygmomanometer. Therefore, BP at rest was estimated in the experiment of Section 4. Of course, if we know the absolute value, we can also calculate the variation from the reference value like the conventional PTT method. So, in this section, we compared the estimation accuracy of the BPV with our proposed method and the PTT method.

After eliminating trouble cases in measuring, 27 sets of data were remained. Features were extracted from these data and evaluated using leave-one-out cross-validation method. BPV and PTT variation (PTTV) were calculated based on BP and PTT signals in the first minute. As a conventional PTT method, BPV was estimated using a simple regression analysis where BPV was the objective variable and PTTV was the explanatory variable. Then we compared BPV estimated by our proposed method with estimated by the PTT method.

**Figure 10:**
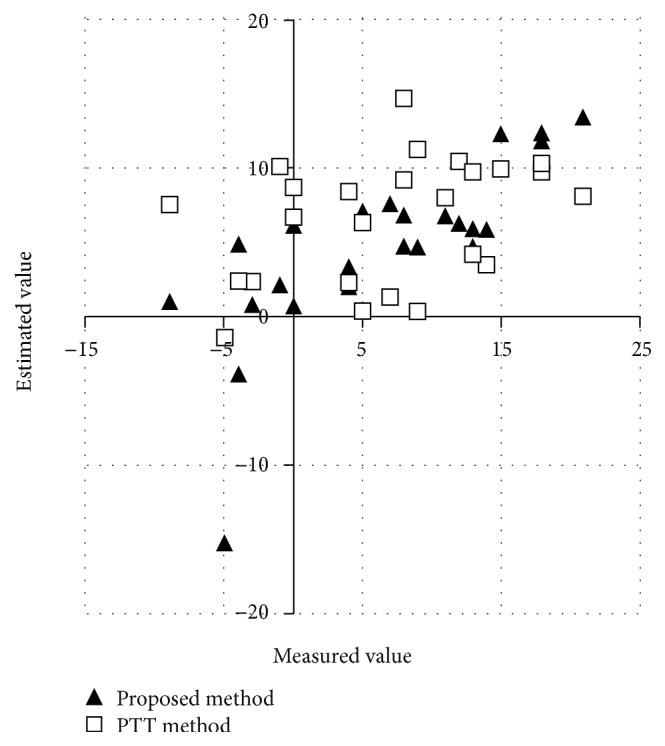
Distribution due to measured value and estimated value by PTT and proposed method.

The estimated result is shown Figure 10 that is a distribution due to measured values and estimated values. As a result by our proposed method, the correlation coefficient between the measured SBPV and the estimated SBPV was 0.73, the mean error was 2.13 mmHg, and the standard deviation of errors was 5.32 mmHg. In contrast, by PTT method, the correlation coefficient was 0.19, the mean error was 0.04 mmHg, and the standard deviation of errors was 7.94 mmHg. Our method could estimate BP more accurately than PTT method.

Next, we considered the contribution of each feature used to estimate BP, especially static features versus dynamic features. When we estimated BP with excluding personal information such as age, sex, and weight by our method, correlation coefficient was 0.88, error mean was 0.08 mmHg, and error standard deviation was 7.0 mmHg. Hence, using static features, that is, personal information, is effective for BP estimation. It was suggested that the basic BP for the subject is estimated by personal information and variation from the basic BP is estimated by PPG features.

## 6. Conclusion

In this paper, we described a monitoring system incorporating a cuffless BP estimation method based on machine learning. In order to collect BP more easily, we used only a wristwatch-type PPG sensor. We implemented a BP estimation system on the cloud server and constructed a system to manage blood pressure and PPG data. In addition, we implemented an application to record BP and life-log at the same time in order to collect the user's life information related to blood pressure. We have developed an application for visualizing the life-log and BP data for the users and the medical staff. Introducing this system has the potential to give appropriate advice to users. In addition, by collecting data, we think that we can identify new factors of hypertension. Our potential future work is the accuracy improvement of BP estimation. Furthermore, we will implement a monitoring system at medical institutions and verify whether medical staff can provide appropriate guidance to patients by collecting data over the long term.

## Figures and Tables

**Table 1:** Features for estimation.

Features	Description
*a*	*a*-wave height
*b*	*b*-wave height
*c*	*c*-wave height
*d*	*d*-wave height
*e*	*e*-wave height
*T*_*a*_	Time elapsing from rise of APG to *a*-wave
*T*_*b*_	Time elapsing from rise of APG to *b*-wave
*T*_*c*_	Time elapsing from rise of APG to *c*-wave
*T*_*d*_	Time elapsing from rise of APG to *d*-wave
*T*_*e*_	Time elapsing from rise of APG to *e*-wave
*b*/*a*	Ratio of *b* to *a*
*c*/*a*	Ratio of *c* to *a*
*d*/*a*	Ratio of *d* to *a*
*e*/*a*	Ratio of *e* to *a*
APG index	(*c* + *d* − *b*)/*a*
PR	Pulse rate counted from the PW of the past 1 min
Height	Height from a preliminary questionnaire [m]
Weight	Weight from a preliminary questionnaire [kg]
Age	Age from a preliminary questionnaire
Sex	Sex from a preliminary questionnaire (male: 0, female: 1)

**Table 2:** Recorded life-log data by the smartphone application.

Method	Timing	Recording data
Manual input	Primary	Height, age
	Once a day	Weight, body temperature
	Each event	Meal (picture, place), bed time hour, awaking hour

Automatic	Constantly	Acceleration (*x*, *y*, *z*), pedometer, GPS, travel distance, calorie
	Each event	Meal (GPS, date)

**Table 3:** Visualized data interface for users.

Content	Description
BPV	BPV (line graph), average of SBP, average of DBP, maximum of SBP and minimum of DBP for the day
BP histogram	Histogram of BP measured during a period of time
BP statistics	Maximum BP and minimum BP for each day in the week
